# TRANSLATION AND CULTURAL ADAPTATION OF THE PREFERENCES FOR EVERYDAY LIVING INVENTORY INTO KOREAN

**DOI:** 10.1093/geroni/igac059.2825

**Published:** 2022-12-20

**Authors:** Jenny Kwon, So Young Shin

**Affiliations:** Miami University, Oxford, Ohio, United States; Inje University, Busan, Pusan-jikhalsi, Republic of Korea

## Abstract

Valid and reliable measures are necessary to provide person-centered care tailored to the individual. However, there are no such measures in Korean nursing home settings. Therefore, the purpose of this study was to translate and culturally adapt the Preferences for Everyday Living Inventory (PELI); test face validity; and finalize a Korean version of PELI (PELI-K). The translation and cultural adaptation were done according to guidelines of the International Society for Pharmacoeconomics and Outcomes. After translating, cognitively capable Korean older adults (n=10) reviewed the PELI items and completed a questionnaire. Face validity was assessed by three questions regarding grammar and wording, understandability, and cultural relevance using a 4-point Likert scale: 1 (strongly disagree) to 4 (strongly agree). Higher scores indicate better validity. The mean (±SD) age of participants was 67 (±2.50) years. The mean score of appropriateness for grammar and wording was 2.70 (±.82); understandability was 3.70 (±.48); and cultural relevance was 3.70 (±.67). Participants found the Korean version of PELI easy to understand and interpret, and culturally relevant. However, some translations have room for improvement in rephrasing sentences and using alternative wording. Based on cognitive debriefing results and several suggestions from participants, necessary changes were made before creating a final version of PELI-K. These findings suggest that PELI was successfully translated and culturally adapted to Korean. Implementing PELI-K in Korean nursing home settings will help with eliciting individual preferences and incorporating them into care delivery. Next steps can evaluate care quality improvement and increased residents’ satisfaction.

